# Sustained in vivo perfusion of a re-endothelialized tissue engineered kidney graft in a human-scale animal model

**DOI:** 10.3389/fbioe.2023.1184408

**Published:** 2023-06-14

**Authors:** Joseph S. Uzarski, Emily C. Beck, Emily E. Russell, Ethan J. Vanderslice, Matthew L. Holzner, Vikram Wadhera, Dylan Adamson, Ron Shapiro, Dominique S. Davidow, Jeff J. Ross, Sander S. Florman

**Affiliations:** ^1^ Miromatrix Medical Inc., Eden Prairie, MN, United States; ^2^ Icahn School of Medicine at Mount Sinai, Recanati/Miller Transplantation Institute, New York, NY, United States

**Keywords:** perfusion, decellularization, recellularization, end stage kidney disease, bioengineered kidney, extracellular matrix, endothelial cells

## Abstract

**Introduction:** Despite progress in whole-organ decellularization and recellularization, maintaining long-term perfusion *in vivo* remains a hurdle to realizing clinical translation of bioengineered kidney grafts. The objectives for the present study were to define a threshold glucose consumption rate (GCR) that could be used to predict *in vivo* graft hemocompatibility and utilize this threshold to assess the *in vivo* performance of clinically relevant decellularized porcine kidney grafts recellularized with human umbilical vein endothelial cells (HUVECs).

**Materials and methods:** Twenty-two porcine kidneys were decellularized and 19 were re-endothelialized using HUVECs. Functional revascularization of control decellularized (*n* = 3) and re-endothelialized porcine kidneys (*n* = 16) was tested using an *ex vivo* porcine blood flow model to define an appropriate metabolic glucose consumption rate (GCR) threshold above which would sustain patent blood flow. Re-endothelialized grafts (*n* = 9) were then transplanted into immunosuppressed pigs with perfusion measured using angiography post-implant and on days 3 and 7 with 3 native kidneys used as controls. Patent recellularized kidney grafts underwent histological analysis following explant.

**Results:** The glucose consumption rate of recellularized kidney grafts reached a peak of 39.9 ± 9.7 mg/h at 21 ± 5 days, at which point the grafts were determined to have sufficient histological vascular coverage with endothelial cells. Based on these results, a minimum glucose consumption rate threshold of 20 mg/h was set. The revascularized kidneys had a mean perfusion percentage of 87.7% ± 10.3%, 80.9% ± 33.1%, and 68.5% ± 38.6% post-reperfusion on Days 0, 3 and 7, respectively. The 3 native kidneys had a mean post-perfusion percentage of 98.4% ± 1.6%. These results were not statistically significant.

**Conclusion:** This study is the first to demonstrate that human-scale bioengineered porcine kidney grafts developed via perfusion decellularization and subsequent re-endothelialization using HUVEC can maintain patency with consistent blood flow for up to 7 days *in vivo*. These results lay the foundation for future research to produce human-scale recellularized kidney grafts for transplantation.

## 1 Introduction

Every year nearly 125,000 people in the United States are diagnosed with end-stage renal disease (ESRD) and join the 800,000 Americans who suffer with the disease (United States Renal Data System, 2022). These patients require chronic dialysis or a kidney transplant for survival, with kidney transplantation currently being the only potential curative option. While there is a consistently higher 5-year survival rate and a better quality of life associated with transplantation compared to peritoneal or hemodialysis (United States Renal Data System, 2022), research efforts are being made to improve dialysis outcomes with the use of expanded hemodialysis (HDx) therapy designed to improve clearance of middle-sized uremic toxins (Catar et al., 2021) and limit remodeling of the peritoneum to increase the duration of peritoneal dialysis therapy Catar et al., 2022).

Currently, the shortage of donor kidney grafts limits the number of renal transplants that can be performed. Despite approximately 24,000 kidney transplants being performed annually, nearly 100,000 Americans remain on the waiting list (Lentine et al., 2022). Even with the expansion of living organ donation programs and extended criteria for the use of deceased donor organs, there is an insufficient supply of kidney grafts available for the increasing number of patients who are diagnosed with ESRD.

The development of a clinically viable bioengineered kidney offers the opportunity to overcome the shortage of donor organs and the need for dialysis for patients with ESRD. Unfortunately, a bioengineered kidney remains a substantial tissue engineering challenge due to this organ’s complex microarchitecture and physiology. Meaningful progress has recently been made in the directed differentiation of pluripotent stem cells into kidney organoids, and several published protocols describe production of kidney organoids containing nephrons and collecting duct structures (Takasato et al., 2016; Morizane and Bonventre, 2017; Wu et al., 2018; Miyoshi et al., 2020) However, while organoid engineering has tremendous potential for disease modeling (Kim et al., 2017), diagnostic drug toxicity screening (Morizane and Bonventre, 2017), gene editing (Kim et al., 2017) and other applications (Miyoshi et al., 2020), organoids developed using these protocols lack a perfusable vasculature and a urinary drainage system. The absence of these structures limits organoid diameter to 1–2 cm and prevents them from recapitulating renal filtration and excretory functions, and therefore precludes their translation to clinical therapies (Miyoshi et al., 2020).

Perfusion decellularization of intact organs enables development of a scaffold that retains the extracellular matrix architecture and composition of the vasculature and urinary drainage system that is three-dimensional (3D), acellular, and human-scale (Ott et al., 2008). This technology has been successfully applied to create a variety of whole-organ scaffolds including heart (Ott et al., 2008), lung (Ott HC et al., 2010; Petersen et al., 2010; Gilpin and Ott, 2015), liver (Uygun BE et al., 2010; Wang et al., 2015), pancreas (Goh et al., 2013), and kidney (Ross et al., 2009; Nakayama et al., 2010; Orlando et al., 2012; Song et al., 2013; Bonandrini et al., 2014; Yu et al., 2014; Caralt et al., 2015; Peloso et al., 2015; Leuning et al., 2019). Promising findings have shown that decellularized kidney grafts can be recellularized and even promote differentiation of pluripotent stem cells along a renal lineage (Ross et al., 2009; Nakayama et al., 2010; Bonandrini et al., 2014; Abolbashari et al., 2016), however, there are limited published reports describing transplantation of these grafts in preclinical models.

The difficulty with sustaining function with decellularized animal kidneys has been reported by several authors. A study of acellular porcine kidney grafts implanted into pigs demonstrated reperfusion with sustained blood pressure without blood extravasation during surgery but complete vascular thrombosis upon histological examination at 2 weeks (Orlando et al., 2012). Another study found that decellularized kidney grafts were completely thrombosed 7 days after implantation into a rat model (Peloso et al., 2015). Separate research overcame this acute thrombogenic response using whole rat kidneys that were decellularized and then recellularized with both rat epithelial and human endothelial cells followed by perfusion in a bioreactor where they produced rudimentary filtrate *in vitro* (Song et al., 2013). When implanted acutely in rats, the grafts showed no signs of thrombus formation, highlighting the importance of re-endothelializing acellular grafts. The authors did not report the length of time these grafts were studied *in vivo*. These results were also achieved in a small animal model which is on a significantly smaller scale compared to human renal grafts.

Prior to the present study, the longest reported sustained perfusion of blood in a recellularized kidney graft was an *in vitro* study performed with porcine whole blood where the grafts were reported to be patent for 24 h (Zambon et al., 2018). Based on our previous research where we demonstrated that continued glucose consumption was a marker for a high rate of re-endothelization in decellularized porcine liver grafts (Mao et al., 2017), we hypothesized that glucose consumption by the recellularized kidney graft would also serve as a marker for its extent of endothelialization and indicate the ability of a recellularized porcine kidney graft to remain patent.

To create a foundation for eventual transplantation of parenchymal-seeded grafts, the present study was conducted to focus on endothelialization of the renal vasculature of decellularized porcine kidneys with human vascular cells followed by the demonstration of sustained perfusion. HUVECs were chosen for kidney revascularization due to the ability to create an HLA bank, favorable proliferation kinetics, phenotypic plasticity with the ability to form fenestrations *in vitro* (Hamilton et al., 2007), and past success with HUVECs for liver recellularization (Shaheen et al., 2020). In conjunction with the above, a key objective for the study included defining a threshold glucose consumption rate (GCR) that could be used to predict *in vivo* graft hemocompatibility and then utilize this threshold to assess the *in vivo* performance of clinically relevant decellularized porcine kidney grafts recellularized with human endothelial cells in an orthotopic transplantation model. Therefore, the following study is limited to patency assessment only and did not assess standard kidney functions like glomerular filtration rate (GFR), which require additional kidney parenchymal cells.

## 2 Materials and methods

The present study was carried out in the facilities of American Preclinical Services (Minneapolis, MN), an Association for Assessment and Accreditation of Laboratory Animal Care (AAALAC) approved facility. The study protocol was reviewed and approved by the facility’s Institutional Animal Care and Use Committee (IACUC). All animals were housed, fed, cared for, and euthanized by study personnel and animal husbandry staff of American Preclinical Services.

### 2.1 Donor kidney selection and recovery

**FIGURE 1:**
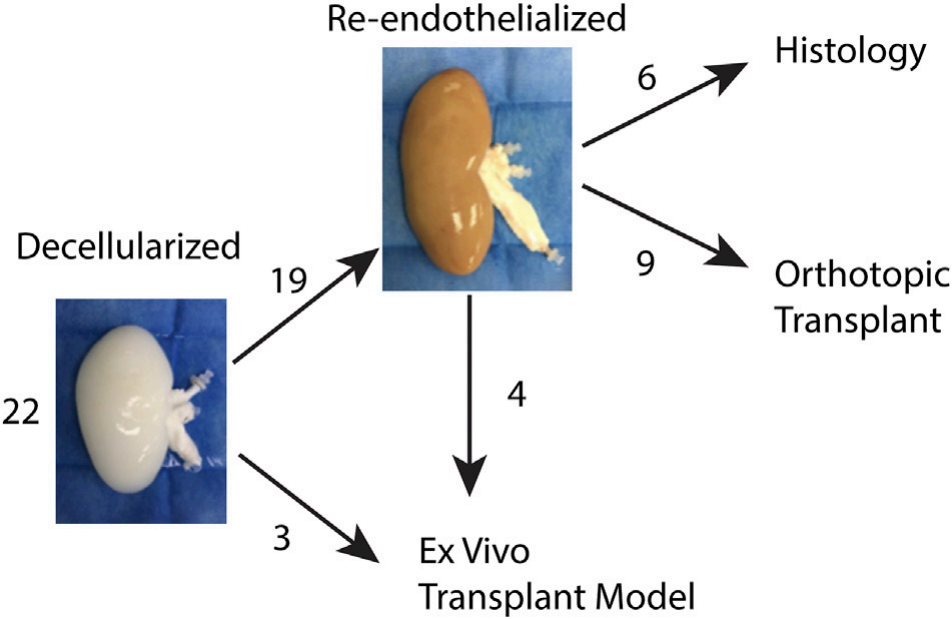
Disposition of harvested porcine kidneys.

Twenty-two porcine kidneys weighing 250–300 g were obtained from adult (approximately 6 months old) male and female Landrace/Yorkshire/Duroc crossbreed pigs purchased from Midwest Research Swine (Glencoe, MN). After recovery, the kidneys were rinsed in saline, bagged, and transported on ice to a separate facility for processing. Figure 1 details the disposition of each of the harvested kidneys for the steps outlined below.

### 2.2 Decellularization

Prior to decellularization, the kidney pairs were removed from ice and passed into a class 10,000 clean room where they were disinfected with peracetic acid (PAA) before and after cannulation. The renal artery, renal vein, and ureter on each kidney were cannulated using appropriately sized polypropylene cannulae (Value Plastics, Loveland, CO) and secured with 3–0 nylon sutures (eSutures, Mokena, IL). Cannulated kidneys were perfusion decellularized using Triton X-100 for 3 h followed by 0.3% sodium dodecyl sulfate (SDS) overnight. The kidneys were then flushed with phosphate buffered saline (PBS) and underwent further disinfection using 1,000 ppm peracetic acid prior to a final PBS flush and packaging. Perfusion pressure was held at a constant value (renal artery: 60 mmHg; renal vein: 40 mmHg; ureter: 20 mmHg) by altering the volumetric flow rate of the peristaltic pump. Flow rates on the artery and vein were consistently below 200 mL/min and flow rate on the ureter was consistently below 100 mL/min. Perfusion alternated between the renal artery, vein, and ureter at evenly spaced intervals during the PAA and PBS stages. The decellularized kidney scaffolds were then packaged with PBS and stored at 4°C for up to 6 months.

### 2.3 Endothelial culture and seeding

**FIGURE 2:**
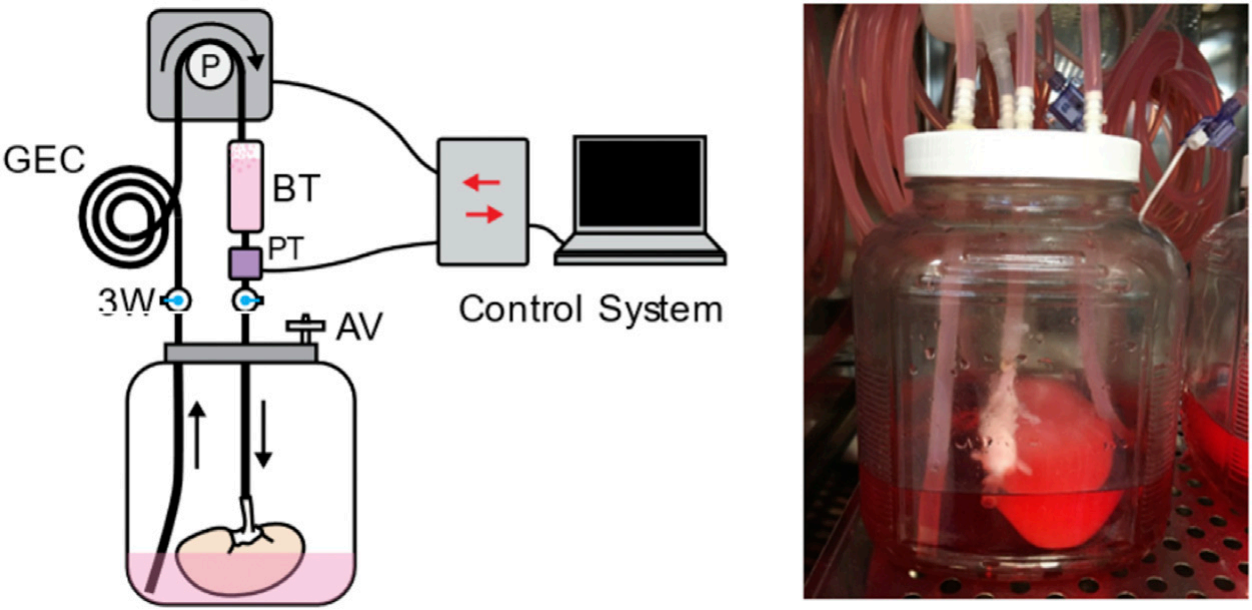
The perfusion system used for kidney recellularization. The system consisted of a reservoir holding the kidney, an air vent (AV), a pressure transducer (PT) to monitor perfusion pressure, a gas exchange coil (GEC) to allow for diffusion of gas into the perfusate, a bubble trap (BT), and several three-way (3W) stopcocks to direct the flow of the media to the kidney. Perfusion was driven by a peristaltic pump under control by custom perfusion software.

To establish thromboresistance for the porcine extracellular matrix and prepare the decellularized kidney grafts for transplantation, human umbilical vein endothelial cell (HUVEC) suspensions were infused sequentially through the renal vein and artery using a perfusion bioreactor system (Figure 2). The bioreactor system used custom perfusion software and a peristaltic pump to drive the flow of culture media into the kidney through either the renal vein or artery, while maintaining the volumetric flow rate or pressure at a specified value. This system enabled more consistent recellularization among different donor kidneys and across different recellularization lots by closely regulating environmental parameters (e.g., perfusion pressure/flow, temperature, pH, and oxygen tension) during seeding and subsequent perfusion culture.

The decellularized kidneys were mounted in the sterile bioreactors inside humidified incubators. They were then perfused through the renal vein at 100 mL/min with a pressure less than 20 mmHg in custom antibiotic-free endothelial media containing endothelial base media (R&D Systems, Minneapolis, MN, #CUST01707), sodium bicarbonate (Sigma-Aldrich, St. Louis, MO, #S5761), fetal bovine serum (FBS) (Fisher Scientific), ascorbic acid (Amresco, Solon, OH, #0764), hydrocortisone (Tocris, Minneapolis, MN, #4093), fibroblast growth factor (FGF) (R&D Systems, #233-FB/CF), vascular endothelial growth factor (VEGF) (R&D Systems, #293-FB/CF), epidermal growth factor (EGF) (R&D Systems, #236-EG), recombinant insulin-like growth factor (R3-IGF) (Sigma Aldrich, #85580C), heparin (Sigma Aldrich, #H3393), and acetic acid (Sigma Aldrich, #A6283) for at least 3 days to confirm sterility. The media was replaced with endothelial media containing 1% penicillin/streptomycin prior to seeding. Primary cryopreserved HUVECs were expanded in 5-chamber CellSTACK cell culture chambers (Corning, Glendale, AZ) using antibiotic-free endothelial media and were lifted for seeding at passage 5–8 (Lonza, Walkersville, MD). After lifting, HUVECs were passed through a 70 µm strainer to remove any cell clumps and were resuspended at a concentration of 1 million cells/mL. Cells were seeded through the vein with 100 mL cell suspension without perfusion for 1 h and then 50 mL cell suspension was injected into the venous perfusion flow field at 50 mL/min. After 24 h, perfusion was switched from the vein to the artery, the media was changed, and another 150 million HUVEC cells were seeded through the artery using the same protocol. At 24 h after the first arterial seeding, the media was replaced, and the flow was increased to 100 mL/min. Metabolite levels in bioreactor media samples were measured daily using a BioProfile FLEX Analyzer (Nova Biomedical, Waltham, MA). Media was changed on alternating days and GCR was monitored. Between 1 and 6 days after the first arterial seeding, the kidney grafts were seeded through the artery a second time using the previously described arterial seeding protocol. The media was changed 24 h after the serial arterial seeding and then the frequency and volume of media changes was adjusted as needed to prevent total glucose depletion.

### 2.4 Cell metabolism

Cell metabolic activity was monitored daily by analyzing a bioreactor media sample. Sample metabolite concentrations were obtained from a Cedex Bio HT Analyzer (Roche) to monitor metabolism of glucose, glutamine, glutamate, ammonia, and lactate dehydrogenase (LDH). When glucose levels fell below 0.2 g/L or ammonia levels rose above 1 mM, additional media was added to the bioreactor reservoir.

### 2.5 Acute *ex vivo* transplant model

**FIGURE 3:**
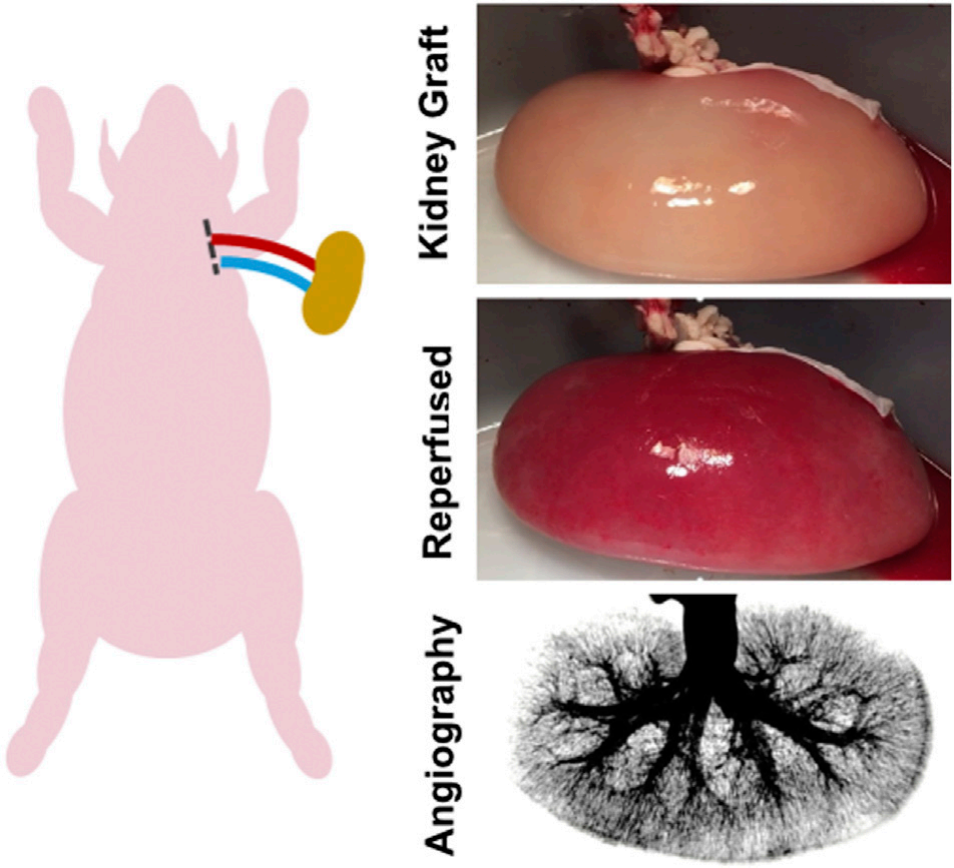
*Ex vivo* transplant model. An *ex vivo* blood loop was created by placing catheters in the carotid artery and jugular vein of an anesthetized adult pig. The catheter lines were connected via Luer adapters to the renal artery and vein of the endothelialized kidney graft.

Functional revascularization of control decellularized porcine kidneys (*n* = 3) and re-endothelialized kidneys (*n* = 4) was tested using an acute *ex vivo* transplant model. This enabled perfusion of the kidneys under physiological hemodynamic conditions in an observable setting (Figure 3). This blood loop system was used for evaluation of vascular integrity, consistency of tissue perfusion, and distribution of flow across the renal vasculature. Catheters were placed into the carotid artery and jugular vein in anesthetized adult Yorkshire Cross pigs weighing 80–90 kg. Receiving intravenous heparin injections to maintain activated clotting time between 175 and 225 s. Tygon tubing and barbed Luer adapters were used to create a loop linking the carotid artery and jugular vein. A Transonic flow meter and 8 mm flow probe (T402 modular research console, Transonic Systems Inc., Ithaca, NY) and pressure T were integrated in the system downstream from carotid access and upstream from the graft. Baseline flow and graft inflow were recorded, and angiographies performed over time.

### 2.6 Bioengineered kidney graft implantation

Following confirmation of functional patency using the *ex vivo* transplant model, functional revascularization was evaluated in a porcine orthotopic kidney transplantation model. Although heterotopic kidney transplantation is the clinical standard, an orthotopic model was chosen here to create a larger surgical field for placement of the endothelialized kidney grafts and to enable graft repositioning to ensure sufficient renal blood flow. Over the course of the nine implants, adjustments were made to the surgical procedure to improve the animal model, which are noted below and in Table 1.

**TABLE 1:** Surgical details and outcomes.

Animal #	Arterial anastomosis	Venous anastomosis	Sling material	Clopidogrel & aspirin administered	Days between arterial HUVEC seedings	Peak GCR (mg/hr)	End GCR (mg/hr)	Days of 3D culture before implant	Patent at POD 7?
1	Renal artery	Renal vein	MIROMESH	No	6	39.54	34.16	22	Yes
2	Renal artery	Renal vein	MIROMESH	No	2	29.95	29.95	27	No—Thrombosed
3	Renal artery	Renal vein	MIROMESH	No	5	36.51	36.51	29	No—Thrombosed
4	Abdominal aorta	Renal vein	MIROMESH	No	1	41.01	41.01	21	No—Terminated POD0 due to uncontrolled graft bleeding
5	Renal artery	Renal vein	Peritoneum	Daily starting POD1	1	30.25	27.09	29	Yes
6	Renal artery	Renal vein	Peritoneum	Daily starting POD1	2	37.92	31.91	28	Yes
7	Renal artery	Inferior vena cava	Peritoneum	Daily starting POD1	1	33.67	33.66	28	Yes
8	Renal artery	Renal vein	Peritoneum	Daily starting POD1	2	43.00	41.20	25	No—Thrombosed
9	Abdominal aorta	Renal vein	None (graft implanted retroperitoneally)	Daily starting POD1	1	41.91	36.20	19	Yes

GCR = glucose consumption rate; POD = post-operative day.

Nine kidney grafts were implanted in separate Yorkshire Cross pigs weighing 75–95 kg. Implantation occurred 19–29 days after initial HUVEC seeding depending on achievement of the targeted GCR (Table 1). Prior to implantation, the kidney grafts were flushed with heparinized saline and the ureter was ligated. Each kidney was spray coated with 2 mL Tisseel (Baxter, Deerfield, IL) using the EASYSPRAY Set (Baxter) and the Tisseel was allowed to set for 3 min. Under general anesthesia and heparin administered to raise the activated clotting time to 175–225 s, the kidney grafts were implanted into pigs. Following a laparotomy, surgeons performed a splenectomy to mitigate acute rejection of the HUVECs, and a nephrectomy. The revascularized grafts were anastomosed to the renal vein and artery or by a side anastomosis to the abdominal aorta and inferior vena cava. The graft was secured to the abdominal wall using a biological sling. MIROMESH Biological Matrix (Miromatrix Medical, Eden Prairie, MN) was used as the sling material in the first 4 cases before switching to the native peritoneum to obtain better stability in remaining cases after graft movement and thrombosis was observed. Vital signs and arterial flow to the kidney were monitored using the Transonic flow meter until the flow stabilized.

The recellularized implanted kidney grafts were monitored with angiography post-operatively and at days 3 and 7. Two of the native kidneys were also monitored at similar timepoints. Daily medications administered included aspirin and ketoprofen for pain management, ceftiofur sodium and ceftiofur crystalline free acid for infection prophylaxis, and methylprednisolone to mitigate immunological rejection of HUVECs. After observing localized vascular thrombosis at the anastomosis site in 2 implanted grafts at days 3 and 7, daily clopidogrel anticoagulation therapy was administered to subsequent recipient pigs starting on post-operative day 1.

### 2.7 Angiography

Angiography was used to assess kidney graft patency following implantation. A sheath was placed in the carotid or femoral artery and a catheter advanced to the location of arterial blood supply of the graft. Omnipaque 300 contrast agent (GE Healthcare, Chicago, IL) was then injected via the catheter. The contrast agent was injected with subtracted angiography using the Pie Medical Software on the Siemens Leonardo Workstation with a Siemens Artis instrument, and images were recorded until the contrast had completely cleared from the kidney. Angiography images were acquired at the frame at which the maximum loading of contrast was retained in the kidney. Using ImageJ, a grayscale histogram of pixels was obtained for the entire region containing the kidney and the pixels with the same grayscale value as the background surrounding the kidney were subtracted. Percent perfusion was then calculated as the percentage of remaining number of pixels retained within the kidney histogram compared to the original number of pixels prior to background subtraction. Results are presented as percent graft perfusion. Following the same procedure, contralateral native kidneys were analyzed in 3 of the recipient pigs to compare to the patency of the kidney grafts.

### 2.8 Histology

Histologic samples were embedded in paraffin, sectioned at 5 µm thickness, and were stained with hematoxylin and eosin (H&E) and Masson’s Trichrome staining procedures (Scientific Solutions, LLC). Imaging was performed using Zeiss Zen software and Axiocam 105 color brightfield microscope camera. For explanted patent kidney grafts, the pigs were euthanized with intravenous barbiturate and the graft was excised and flushed with 300 mL PBS followed by 300 mL 10% neutral buffered formalin (NBF, VWR). If the kidneys could not be fully flushed due to thrombosis, they were cut into 1 cm slices and submerged in 10% NBF to enable further processing similar to the patent kidneys. Regions (1 cm thick) were cut from the upper and lower pole as well as the midline of the kidney and were submerged in 10% NBF for 2 days.

### 2.9 Immunofluorescence staining

Paraffin embedded sections (Scientific Solutions, Fridlay, MN) were deparaffinized, rehydrated, and subjected to antigen retrieval in a Decloaking Chamber NxGen (Biocare Medical, Pacheco, CA). Slides were washed with sodium borohydride in PBS to reduce autofluorescent signal from red blood cells. They were then co-stained with appropriate primary and secondary antibodies and DAPI, then mounted using ProLong Diamond Antifade Mountant (Thermo Fisher Scientific). Imaging was performed using Jenoptik Gryphax software and Progres Gryphax fluorescent microscope camera. Anti-CD31 antibody (Abcam, Freemont, CA, #ab28364) is reactive to both human and pig endothelial cells. Anti-CD31 antibody (Abcam, #ab187377) is reactive to only human endothelial cells. A co-stain with these antibodies was used to differentiate between HUVEC and recipient pig endothelial cells. Anti-VE-cadherin antibody (Abcam, # ab33168) was also used to identify both human and pig endothelial cells. Anti-human nucleoli antibody (Abcam, # ab190710) was used to identify human endothelial cells. Anti-Collagen I antibody (Abcam, #ab34710), Anti-Collagen IV antibody (Abcam, #ab6586), and Anti-Laminin antibody (Abcam, #ab11575) were used for matrix characterization. AlexaFluor 488 (Thermo, #A11034, #A11029) and 555 secondary antibodies (Thermo, #A21429, #A21424) were used to detect the primary antibodies.

### 2.10 Statistics

A Kruskal-Wallis ANOVA was performed on the angiography data using GraphPad Prism 8.1.1 software (GraphPad Software, Inc., La Jolla, CA), where *p* ≤ 0.05 was considered significant. Data are reported as mean ± standard deviation.

## 3 Results

**FIGURE 4:**
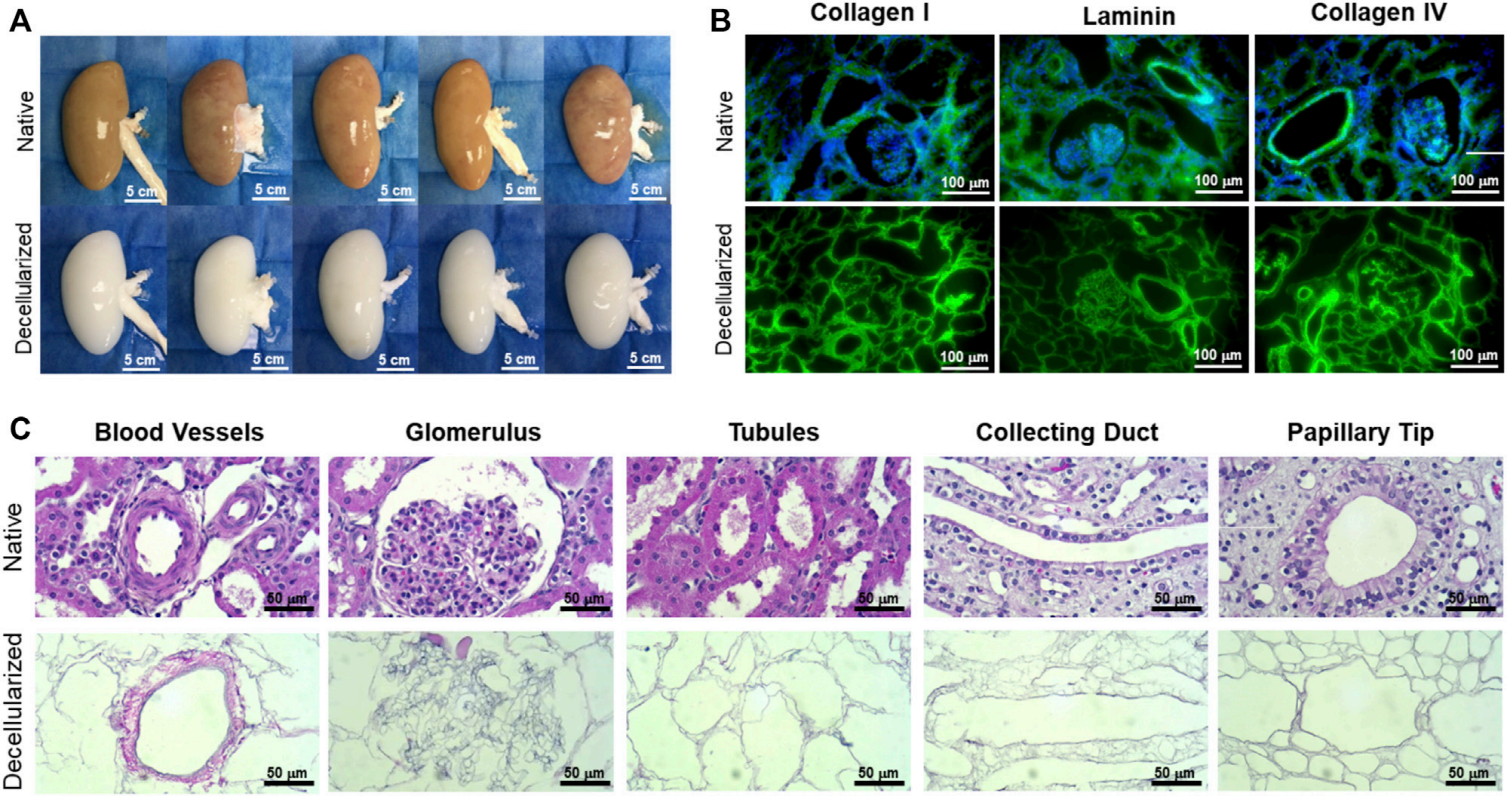
Representative gross, immunofluorescence, and histological images comparing native and decellularized kidneys. **(A)** Decellularization caused a gross loss of color as the native porcine cells were solubilized and extracted, while the original size, shape, and structure of the kidney were well-retained in the decellularized scaffold (scale bars, 5 cm). **(B)** Immunofluorescence staining showed retention of collagen I, laminin, and collagen IV (green) and complete removal of nuclei (blue DAPI stain) (scale bars, 100 µm). **(C)** High-magnification H&E images revealed the ECM structures left behind in decellularized kidneys, including blood vessels, glomeruli, nephron tubules, collecting ducts, and papillae (scale bars, 50 µm).

The kidneys obtained from adult donor pigs retained their native gross shape, size, and ECM proteins, including collagen I, laminin, and collagen IV following decellularization (Figures 4A, B). The resulting matrix retained the appropriate 3D architecture for renal microstructures, including blood vessels, glomeruli, nephron tubules, collecting ducts, and papillae (Figure 4C). Sixteen kidneys were used for the study to determine the GCR threshold and 9 were used to assess the vascular patency of implanted re-endothelialized kidney grafts.

### 3.1 Determination of threshold glucose consumption rate (GCR)

**FIGURE 5:**
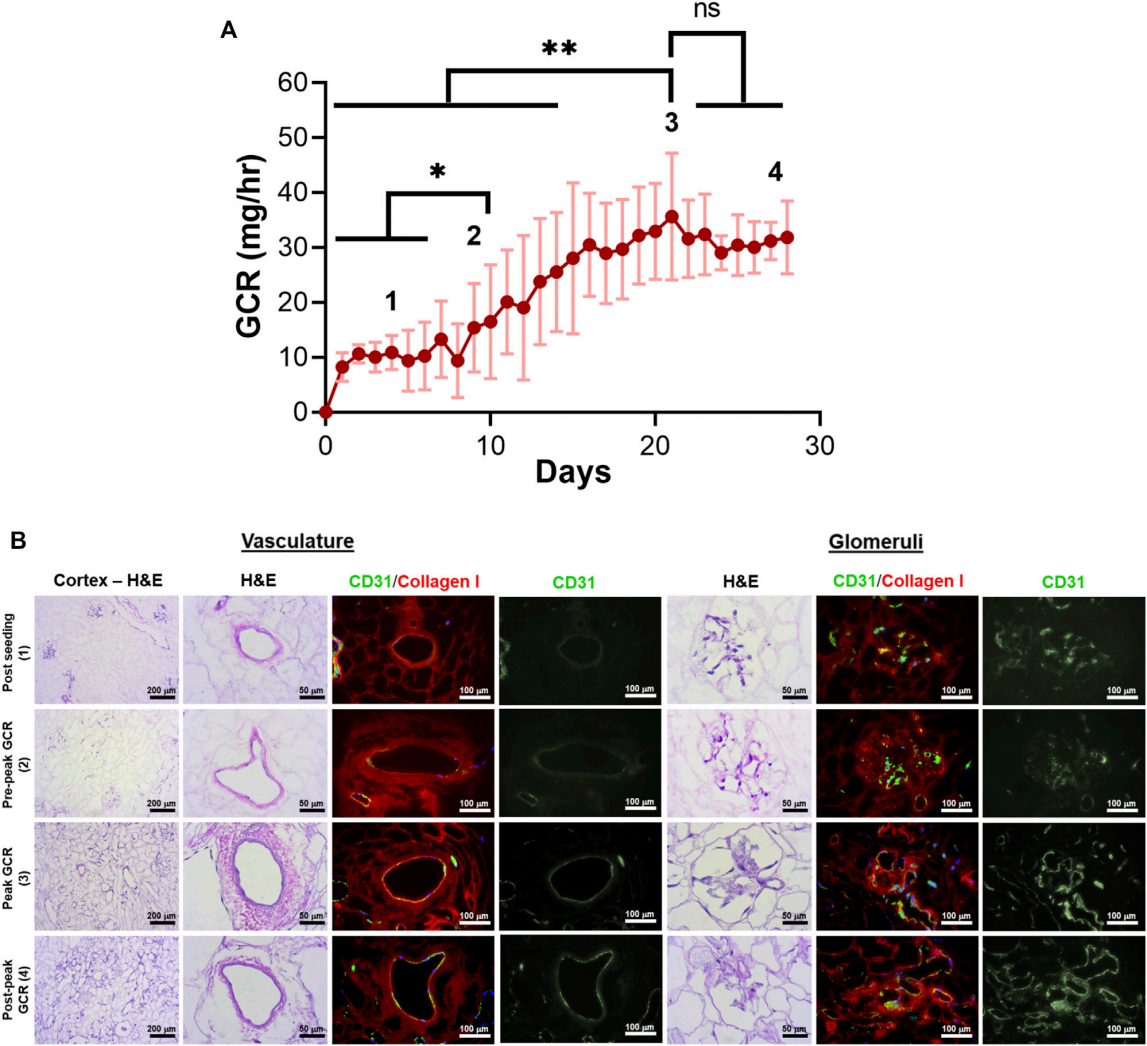
Correlation of glucose consumption kinetics with vascular endothelial cell coverage for decellularized porcine kidneys reendothelialized with HUVECs. **(A)** Total glucose consumption rate (GCR) by endothelial cells was calculated by monitoring the changes in glucose concentration in bioreactor media samples over time. Four distinct zones (1 through 4) were defined to distinguish endothelial cell coverage based on GCR and culture time. *p < 0.05, ****p < 0.0001. **(B)** Representative H&E images and immunofluorescence images revealed endothelialization of the vasculature and glomerular capillaries at each zone (green: CD31 staining HUVECs, red: collagen I staining the porcine matrix; blue: DAPI staining cell nuclei).

Daily monitoring of total glucose consumption rate (GCR) by endothelialized grafts demonstrated GCR was a non-destructive indicator of re-endothelialization. GCR remained around 10 mg/h on days 0–7 after seeding (Figure 5A1) before gradually increasing, with a peak mean GCR (39.9 ± 9.7 mg/h) achieved at 21 ± 5 days (Figure 5A3). GCR plateaued after peaking around day 21 of culture (Figure 5A4). The period of increasing GCR leading up to, but still significantly lower than, peak GCR (day 8–14) was defined as pre-peak GCR (Figure 5A2). Mean GCR on day 14 was 25.5 ± 10.9 mg/h.

HUVECs were found to engraft both large vessels and glomerular capillaries early after seeding (Figure 5B, “Post Seeding (1)”). The density of endothelial cells in blood vessels and glomeruli increased along with GCR between days 7–21 [Figure 5B “Pre-peak GCR (2)”, “Peak GCR (3)”). The native porcine vasculature was uniformly reconstituted with HUVECs with all histologic sections evaluated showing near complete circumferential coverage when peak mean GCR was achieved (Figure 5B, “Peak GCR (3)”]. In this peak period, the endothelial cells were evident in large blood vessels and glomerular capillaries.

**FIGURE 6:**
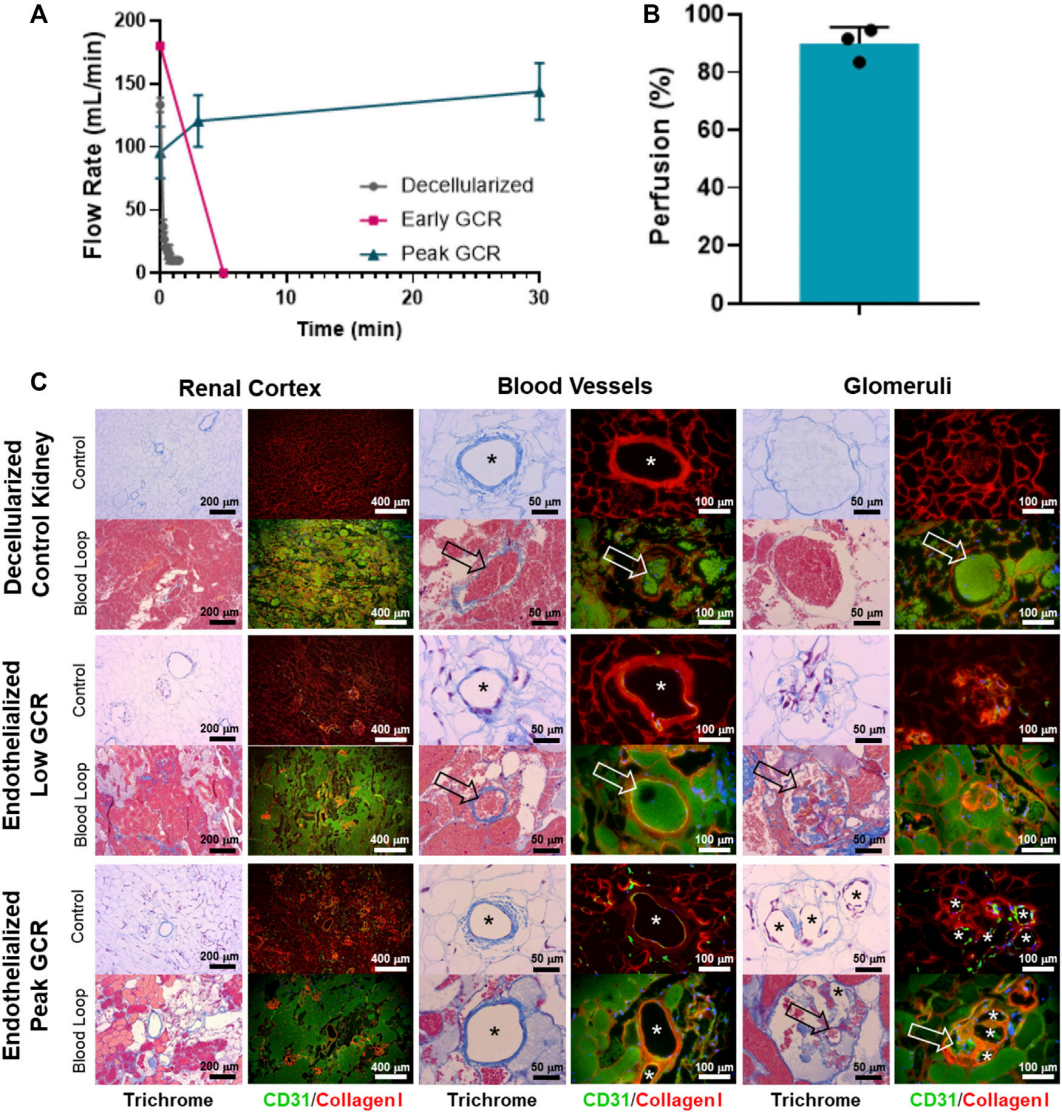
**(A)** A Transonic flow meter was used to monitor volumetric flow rate of blood leading into the renal artery of the organ. Decellularized kidneys (no cells, *n* = 3) and an endothelialized graft with Low GCR (*n* = 1) did not maintain blood flow beyond several minutes. Endothelialized grafts near Peak GCR maintained consistent perfusion throughout the entire study period (30 to 80 minutes, *n* = 3). **(B)** Quantified angiographic images showed a high percentage of graft perfusion. Data for perfusion was reported as mean + standard deviation and with individually plotted data points. **(C)** Representative Masson’s Trichrome stain or CD31/Collagen I immunofluorescence images (asterisks denote the lumens of patent blood vessels; open arrows point to thrombosed blood vessels).

Measurement of perfusion using the acute *ex vivo* transplant model showed the 3 control decellularized kidneys thrombosed rapidly within 5 min of being perfused with the inlet volumetric flow rate rapidly declining to zero (Figures 6A, B). Similar results were seen for the single endothelialized kidney that had a GCR below 20 mg/h (Figures 6A, B) which was associated with incomplete vascular coverage (Figure 5B, “Post Seeding (1)”). In contrast to the above, the 3 endothelialized kidneys expressing Peak GCRs greater than 20 mg/h had sustained, consistent perfusion rates of over 100 mL/min after 30 min of continuous blood perfusion (89.9% ± 5.7% of baseline values). Quantification via angiography stills after a minimum of 30 min confirmed consistently high perfusion percentage of endothelialized grafts (Figure 6B). Histologically, the vasculature in grafts at Peak GCR remained patent and free of thrombus throughout the recellularized kidney after perfusion with blood (Figure 6C). Based on these results, it was determined that a minimum glucose consumption rate threshold of 20 mg/h was required for endothelialized grafts to maintain consistent perfusion in acute (>30 min) *ex vivo* blood loops.

### 3.2 Vascular patency of re-endothelialized kidney grafts

**FIGURE 7:**
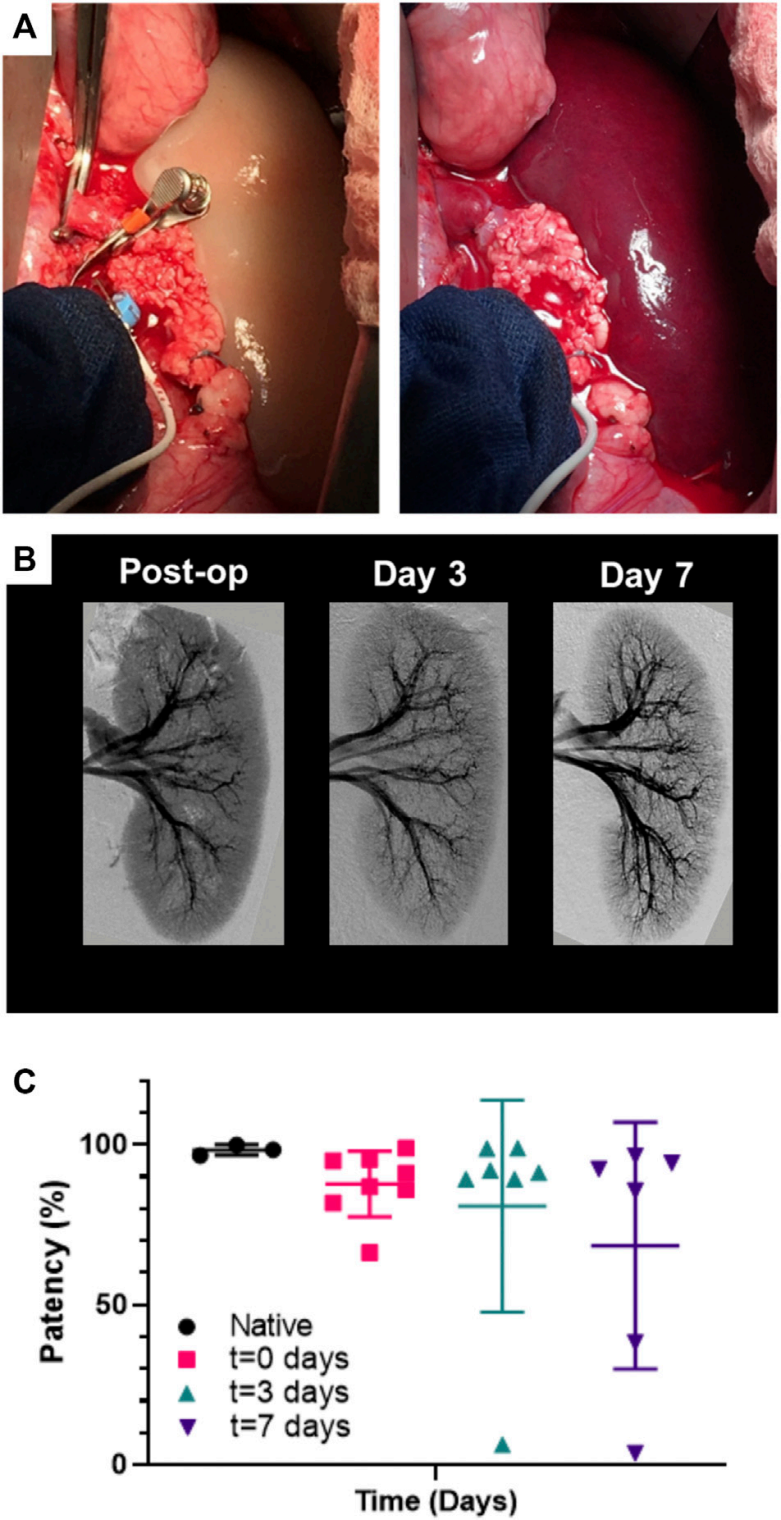
Perfusion of endothelialized kidney grafts. **(A)** Anastomosed kidney graft before and after reperfusion. **(B)** Representative angiography of kidney graft through post-operative day 7. **(C)** Percent perfusion quantified from angiographies performed on the day of surgery through post-operative day 7 for implanted recellularized kidneys (*n* = 9) and control contralateral native kidneys (*n* = 3). Lines represent medians and interquartile ranges.

Table 1 reviews the outcomes of the 9 implanted kidney grafts. Reperfusion was initially uniform for all revascularized kidneys with contrast reaching all the way to the cortical edges of the kidney (Figure 7B; Supplementary Movie S1). One pig died unexpectedly due to excessive peri-operative bleeding and 2 pigs were terminated at days 3 and 7 due to lack of perfusion resulting from thrombosis which was suspected to be a result of graft movement and vessel torsion. Five of the remaining 6 grafts (83.3%) remained patent through 7 days after implantation showing renal perfusion during follow-up angiography. All pigs which developed thrombosis had kidney grafts stabilized by MIROMESH and were not administered clopidogrel or aspirin. Vascular patency was observed in one implant at day 7 in the absence of anti-coagulant therapy before clopidogrel was added to address thrombosis at the anastomoses. The 3 native kidneys had a mean perfusion percentage of 98.4% ± 1.6% compared to 87.7% ± 10.3%, 80.9% ± 33.1%, and 68.5% ± 38.6% for the revascularized kidneys post-reperfusion, on day 3, and on day 7 respectively. These differences were not statistically significant (Figure 7C).

### 3.3 Histologic findings for re-endothelialized kidney grafts

**FIGURE 8:**
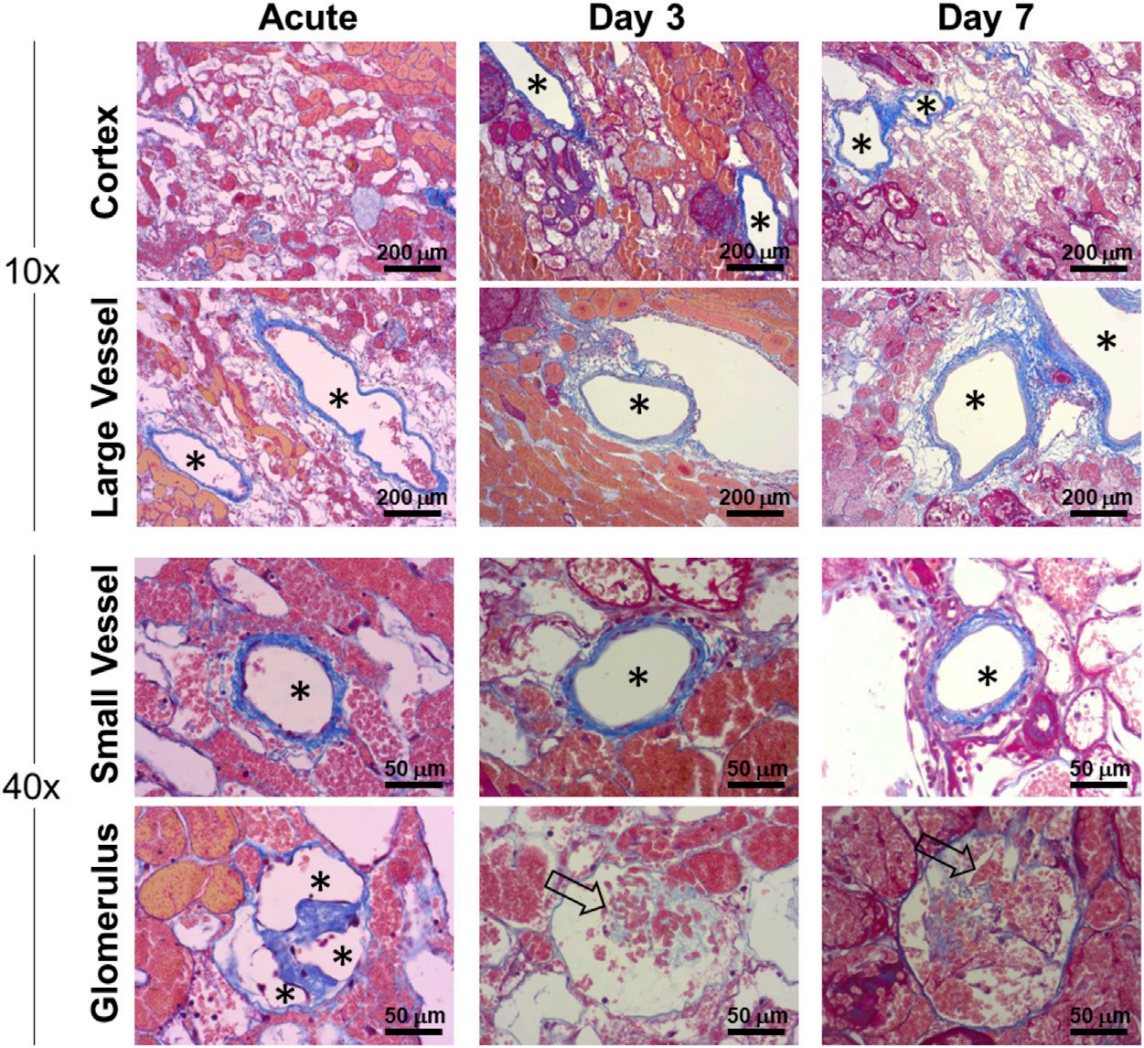
Histological characterization of vascular patency in implanted endothelialized kidney grafts acutely (approximately 2 h post-reperfusion), Day 3 and Day 7. Representative trichrome images show large (>250 µm) and small (<100 µm) blood vessels that remained patent and clear of residual blood after post-explant flushing. Asterisks denote the lumens of patent blood vessels. Open arrows indicate occluded glomerular capillaries.

Patent grafts were explanted for histological analysis at approximately 2 h post-reperfusion (acute) and at Days 3 and 7. The grafts were flushed with saline through the renal artery to remove residual blood before fixation. Histological trichrome staining of the acute explanted kidney showed the vasculature, including glomerular capillaries, remained clear of thrombus (Figure 8). The nephron tubules and surrounding interstitium showed accumulation of clotted blood due to residual vascular leakage that could not drain through the ligated ureter. Glomerular capillaries in grafts explanted at subsequent follow-up time points (Days 3 and 7) showed evidence of occlusion associated with a lack of endothelial lining (Figure 8).

**FIGURE 9:**
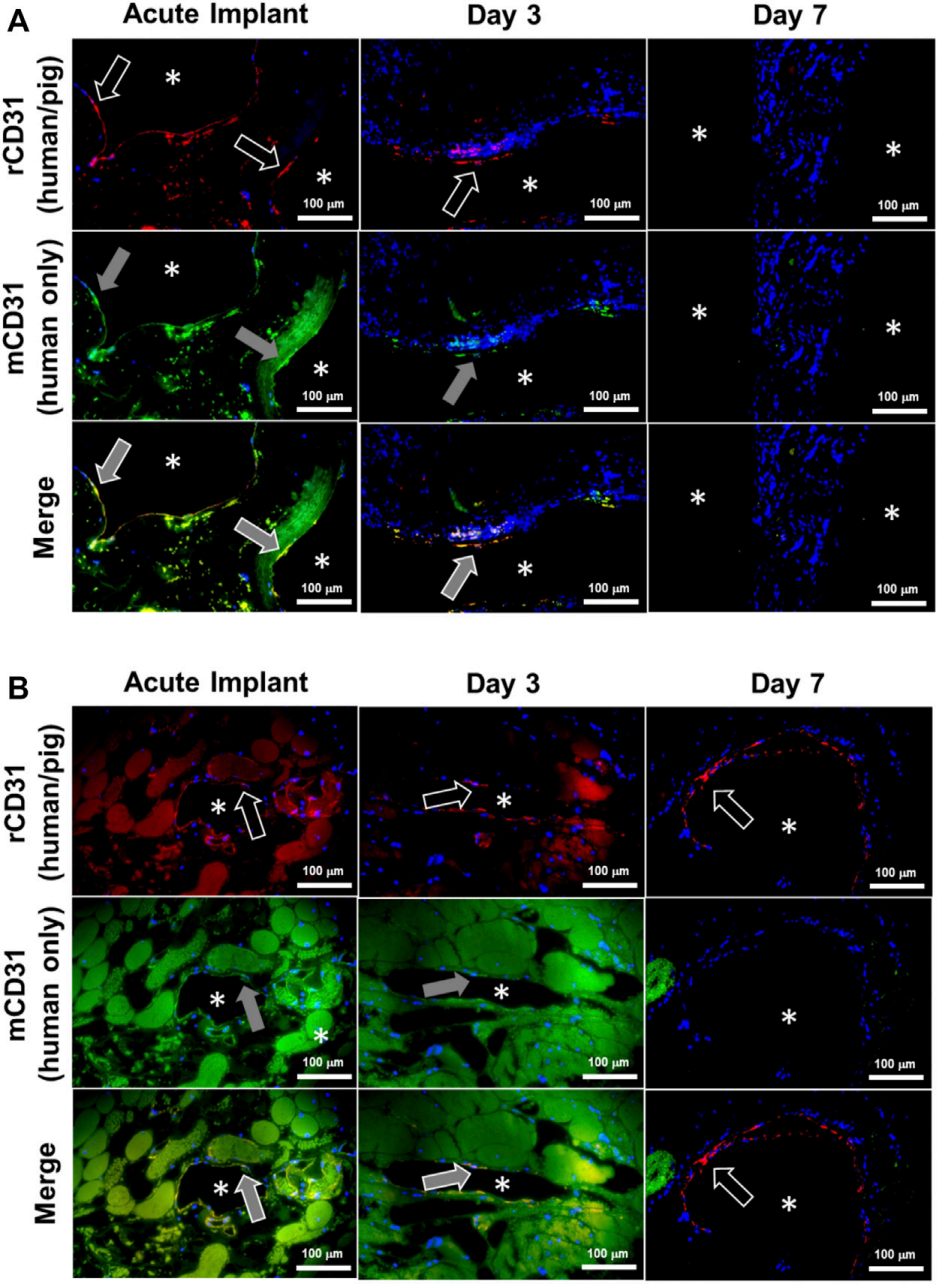
Donor human endothelial cell turnover with recipient porcine endothelial cells in chronically transplanted endothelialized kidney grafts. Endothelial cells were identified as donor (human) or recipient (porcine) in explanted kidney grafts using immunofluorescence staining. Images show the luminal surfaces of **(A)** major blood vessels exceeding 500 µm in diameter, or **(B)** minor blood vessels less than 250 µm in diameter. Asterisks denote the lumens of patent blood vessels. White arrow outline indicates positive reactivity by rabbit CD31 antibody (rCD31; red stain), which reacts to both human and pig endothelial cells. Grey arrow fill indicates positive expression by mouse CD31 antibody (mCD31; green stain), which reacts positively only in human endothelial cells. Top row: rCD31/DAPI overlay. Middle row: mCD31/DAPI overlay. Bottom row: rCD31/mCD31/DAPI merged images. Orange shows colocalization of both antibodies indicating human endothelial cells. Note that the presence of residual blood causes faint background staining in some images. Donor human endothelial cells were completely absent from the graft by Day 7, while new pig endothelialization started to occur at Day 7. Scale bars, 100 µm.

Despite methylprednisolone administration to limit immune rejection of human cells, the HUVECs were observed to gradually disappear from the kidney grafts between post-operative day 3 and 7, and grafts explanted from day 7 were absent of any HUVECs as determined by immunofluorescence staining of fixed tissue (Figures 9A, B; Supplementary Figures S1, S2). Despite this apparent lack of viable human endothelial cells, the vasculature remained patent and the luminal surface did not initiate a clotting cascade, as evidenced by the lack of thrombus (Figures 8, 9). By Day 7 porcine vascular endothelial cells were observed within the renal vasculature starting with the minor blood vessels (Figure 9B).

## 4 Discussion

Perfusion decellularization technology enables the removal of cells and cellular antigens from kidneys with the promise to serve as the scaffold for bioengineering of functional human-scale kidneys and thus, eliminate the need for dialysis and the transplant waiting list (Song et al., 2013; Uzarski et al., 2014; Ott et al., 2008). However, an essential design requirement for a transplantable bioengineered organ is a functional vascular supply that can maintain physiological blood flow without thrombosing. Decellularized organs by definition do not meet this requirement due to widespread exposure of the vascular basement membrane to blood, the proteins of which are highly thrombogenic (Balleisen and Rauterberg, 1980; Willette et al., 1994) and initiate rapid thrombosis even in an animal treated with heparin (Bonandrini et al., 2014; Mao et al., 2017) (Figure 6).

The renal vasculature branches from the renal artery and vein into interlobar vessels down to individual glomerular capillaries and the vasa recta, which are essential to renal function by facilitating reabsorption/excretion via proximity to the intertwined network of nephron tubules (Molema and Aird, 2012). Endothelialized vessels facilitate patent blood flow *in vivo* and therefore are critical to survival of implanted bioengineered organs by contributing to the delivery of oxygen and nutrients to parenchymal cells (Pellegata et al., 2018). We developed our approach to re-endothelialize clinically relevant decellularized porcine scaffolds based on a panel of criteria: 1) adequate histological vascular coverage with endothelial cells, 2) demonstrated increases in cellular metabolism kinetics by non-destructive monitoring to predict patency, and 3) functional hemocompatibility for sustained *in vivo* perfusion. All three criteria were used to refine the methods for endothelial cell seeding, recellularized scaffold perfusion culture, and systematic hemodynamic perfusion to ultimately reach predictive outcomes. Similar to our previous finding that a 10 mg/h minimum GCR for adequate histological endothelial cell coverage and contrast perfusion in recellularized porcine livers (Mao et al., 2017), endothelialized kidney grafts exceeding a minimum GCR threshold of 20 mg/h in the present study maintained persistent perfusion without thrombosis upon exposure to blood flow. The combination of histologic vascular coverage and lack of coagulation supports near-complete re-endothelialization of the vasculature. This rigorous approach allowed for predictability of the readiness of recellularized kidney grafts for transplantation based on non-destructive evaluation (Uzarski et al., 2017).

Differences in human and porcine anatomy required the surgical approach to kidney transplantation used in this study to differ from the standard retroperitoneal access used clinically (Golriz et al., 2012). While human patients are typically positioned supine, retroperitoneal access in the pig requires the animal to be positioned on its side, reducing operating space in the surgical field and making vascular anastomosis challenging. In this study, midline laparotomy performed on supine pigs provided a larger surgical field but allowed positional changes in transplanted grafts after animal recovery that caused the anastomosed vessels to kink or twist, leading to turbulent hemodynamics near the anastomoses. These complications resulted in thrombosis in the renal artery and/or vein, a major source of graft failure in this study. Thrombotic occlusion was mitigated by securing the kidney graft to the abdominal wall using the native peritoneum that stabilized the graft after recovery, thereby prolonging graft patency. In addition, clopidogrel was added to the protocol (starting on post-operative day 1) to specifically reduce the thrombosis observed at the anastomosis sites after an initial 2 kidneys demonstrated continuous perfusion through 1 or 3 days in the absence of anti-coagulation therapy before ultimately succumbing to thrombosis at the anastomoses. Further improvement of the surgical model to overcome these challenges will facilitate heterotopic kidney transplantation in the retroperitoneum, the standard clinical practice. Variability in transplant procedures and timing is a limitation of this study. However, achievement of sustained vascular perfusion *in vivo* in 5 out of 6 kidneys that did not experience vessel kinking and torsion, despite differences in procedures, represents a significant advancement to the field of human-scale kidney recellularization.

Transplanted porcine kidney grafts re-endothelialized with HUVECs maintained vascular patency for up to 1 week post-operatively following *in vivo* orthotopic implantation. Angiographies from the endothelialized grafts performed at regular follow-up intervals showed that patent grafts maintained consistent and uniform perfusion based on gross qualitative visualization (Figure 7; Supplementary Movies S2–S4). Despite administration of steroid immunosuppression therapy, a gradual reduction in HUVEC coverage was observed over time, likely due to an immune response to the human cells despite steroid treatment. Considering the continued graft patency based on angiography which was assessed both qualitatively in real-time and quantitatively post-mortem, it was unexpected that most blood vessels would lose human endothelial cell coverage after 7 days. Initial vascular thromboresistance was conferred by endothelial cells seeded in the grafts as decellularized kidney grafts showed thrombosis within minutes despite the direct use of heparin. While the addition of clopidogrel may have provided some thromboresistance, three initial kidney grafts implanted in animals that did not receive clopidogrel were patent until days 1, 3, and 7 before kinking and/or thrombosis at the anastomosis site, indicating that endothelialized kidneys were capable of sustaining renal blood flow.

The finding that a bioengineered kidney can remain patent during endothelial coverage turnover is potentially promising as the immune system is likely to target endothelial cells even with robust immune suppression strategies, and the vasculature can tolerate exposure of the extracellular matrix until native host endothelial cells are deposited into the graft. The continued patency despite loss of HUVECs from the vasculature suggests a possible novel means for maintenance of patency for the bioengineered kidney grafts. We hypothesize that either the HUVECs are being masked in the implants and are not being detected by immunostaining or that the recellularization with HUVECs leads to an anti-thrombogenic surface following the loss of HUVECs, which lends the surface to gradual revascularization by host endothelial cells. Further studies are needed to understand these results. Further work is also needed to stabilize glomerular capillary endothelialization *in vivo* through the recellularization of the epithelial niche and characterization of fenestrations critical to renal function (Ballermann, 2005). These fenestrations, which HUVECs have been demonstrated to be capable of forming *in vitro*, are critical for glomerular filtration (Hamilton et al., 2007). Future research is also needed to develop a better immunosuppression protocol for this porcine model to mitigate xenogenic cell rejection. It should be noted that the loss of HUVECs is a limitation of placing human cells into an animal model (a reverse xenotransplant) and that this would not be indicative of what would occur when decellularized porcine kidney grafts recellularized with HUVECs is transplanted into a human.

Although the present study demonstrated the ability to predict and maintain graft patency, further studies are needed to assess and document glomerular function and urine production by introducing additional kidney parenchymal cells to the bioengineered kidney grafts. The addition of these parenchymal cells will allow assessment of the glomerular filtration rate, one of the standard functional assessments of human kidneys for transplant (Roemhild et al., 2020). Looking ahead to future potential clinical translation of this approach, a short-term strategy would be to source human cells from donor kidneys that were unable to be placed for transplant (Abdelwahab Elhamahmi et al., 2018). Immunosuppression will still be required for tolerance of bioengineered kidneys produced using this strategy, but the availability of kidneys for transplantation can be increased above current levels. The long-term strategy is to circumvent the need for patient immunosuppression by obtaining autologous cells from the intended transplant recipient using biopsy and/or cellular reprogramming via induced pluripotency/directed differentiation. Recently established protocols detailing directed human induced pluripotent stem cell differentiation into podocytes (Yoshimura Y, et al., 2019), nephron progenitor (Takasato et al., 2016; Morizane and Bonventre, 2017), and ureteric bud collecting duct cells support this approach (Mae et al., 2019). However, considerable development and scale-up work is needed to produce billions of specialized kidney cells required for scaffold recellularization.

## 5 Conclusion

In summary, this study is the first to demonstrate that human-scale bioengineered kidney grafts developed via perfusion decellularization and subsequent re-endothelialization with HUVECs can remain patent with consistent blood flow for up to 7 days *in vivo*. These results provide a basis for further exploratory studies to assess the potential for using bioengineered kidneys as an alternative to human allograft kidneys.

## Data Availability

The original contributions presented in the study are included in the article/Supplementary Material, further inquiries can be directed to the corresponding author.
